# Fast blood flow index reconstruction of diffuse correlation spectroscopy using a back-propagation-free data-driven algorithm

**DOI:** 10.1364/BOE.549363

**Published:** 2025-02-26

**Authors:** Zhenya Zang, Mingliang Pan, Yuanzhe Zhang, David Day Uei Li

**Affiliations:** Department of Biomedical Engineering, University of Strathclyde, 16 Richmond Street, Glasgow, G1 1XQ, United Kingdom

## Abstract

This study introduces a fast and accurate online training method for blood flow index (BFI) and relative BFI (rBFI) reconstruction in diffuse correlation spectroscopy (DCS). We implement rigorous mathematical models to simulate the auto-correlation functions (*g*_2_) for semi-infinite homogeneous and three-layer human brain models. We implemented a fast online training algorithm known as random vector functional link (RVFL) to reconstruct BFI from noisy *g*_2_. We extensively evaluated RVFL regarding both speed and accuracy for training and inference. Moreover, we compared RVFL with extreme learning machine (ELM) architecture, a conventional convolutional neural network (CNN), and three fitting algorithms. Results from semi-infinite and three-layer models indicate that RVFL achieves higher accuracy than the other algorithms, as evidenced by comprehensive metrics. While RVFL offers comparable accuracy to CNNs, it boosts training speeds that are 3900-fold faster and inference speeds that are 19.8-fold faster, enhancing its generalizability across different experimental settings. We also used *g*_2_ from one- and three-layer Monte Carlo (MC)-based *in-silico* simulations, as well as from analytical models, to compare the accuracy and consistency of the results obtained from RVFL and ELM. Furthermore, we discuss how RVFL is more suitable for embedded hardware due to its lower computational complexity than ELM and CNN for training and inference.

## 1. Introduction

Blood flow is a critical clinical indicator, quantitatively reflecting the delivery of nutrients, such as oxygen, and the removal of metabolic waste in biological tissues. Medical imaging techniques such as computed tomography (CT) coronary angiography [1], positron emission tomography (PET) [2], Doppler ultrasound (DU) [3], and magnetic resonance imaging (MRI) [4] provide visual representations of blood flow dynamics. However, these methods often require ionizing radiation and the injection of contrast agents, making continuous clinical monitoring challenging. Diffuse correlation spectroscopy (DCS) [5–7] has emerged as a promising alternative due to its portability, affordability, and capability for continuous monitoring. DCS measures blood flow by characterizing rapid speckle intensity fluctuations caused by the multiple scattering of particles stimulated by coherent near-infrared light. The recorded temporal photon intensity signal is converted into the normalized intensity autocorrelation function $g_{2}$ during an acquisition window. Single-photon detectors [8–10] have been employed due to their high frame rate and high signal-to-noise ratio (SNR). Blood flow index (BFI, mm^2^/s) is a related parameter of the intensity fluctuations. A semi-infinite homogeneous diffusion model has been adopted to extract the BFI from living tissues [6,11,12] and a liquid phantom [13,14] due to its simplicity. While the homogeneous model generates data quickly and is accurate for homogeneous media, it is unsuitable for multi-layered tissues, such as the human brain, where photons interact with superficial layers. Photon interaction in superficial layers contradicts the model’s inherent assumption of tissue homogeneity.

Although the human brain is usually categorized into five-layer slabs—scalp, skull, cerebrospinal fluid (CSF), gray matter, and white matter—the three-layer model effectively approximates these five layers by merging the CSF, gray matter, and white matter into one layer [15]. Zhao *et al.* [15] utilized a three-layer analytical model to generate noiseless $g_{2}$ and employed a nonlinear least squares fitting (NLSF) algorithm (*fminsearchbnd()* in MATLAB) to investigate the errors in reconstructing the scalp and cerebral blood flow index (SBFI and CBFI), affected by varying physiological and tissue optical parameters. A CNN-based approach was used to [16] compare the fitting method with noisy $g_{2}$ generated using the same analytical model. Both studies used Monte Carlo (MC) simulations to generate independent data for evaluation, where the CNN-based approach demonstrated higher accuracy than the fitting methods in terms of absolute and relative BFI (rBFI). NLSF methods require prior knowledge of optical parameters, making accuracy sensitive to the initialization of these parameters [15]. Although deep neural networks (DNNs) have emerged in DCS research [16–20], their accuracy is compromised for new data due to the dependency on the optical properties of the tissue in a specific setup. Even with transfer learning [14], it still takes minutes to re-train DNNs on high-performance GPUs, which hinders real-time monitoring in clinical settings.

To address these challenges, we implemented a single-hidden-layer forward network (SLFN), known as random vector functional link (RVFL), designed to maintain high accuracy while eliminating the need for slow re-training. This approach is back-propagation-free and allows for real-time training. Additionally, training data generation is expedited using analytical models for both the semi-infinite and three-layer human brain models. Unlike existing work that compares against only one fitting algorithm, we comprehensively evaluated RVFL’s performance against a back-propagation-free extreme learning machine (ELM), three iteration-based NLSF methods, and a CNN architecture.

**Fig. 1.**
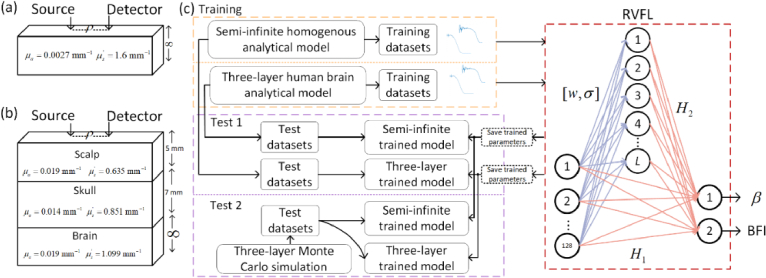
(a) and (b) depict examples of the geometries of the target semi-infinite and three-layer human brain media, with the optical parameters serving as examples from our evaluation cases. (c) illustrates one training and two test pipelines for both semi-infinite and three-layer models. The topology of the RVFL is highlighted in the red dashed box, and the variable annotations are explained in Section 3. Trained parameters (indicated by black dashed boxes) for the analytical model-generated semi-infinite and three-layer datasets are saved and used for performance evaluation. These trained parameters were also used for independent testing on Monte Carlo-generated datasets.

Figure 1 illustrates the framework of this work. The semi-infinite homogeneous and three-layer models for continuous-wave diffuse correlation spectroscopy (CW-DCS), shown in Fig. 1(a) and Fig. 1(b), are utilized for data generation and the implementation of RVFL in Section 3. The performance assessment is detailed in Section 4.

## 2. Prior work

### 2.1. Propagation-free training algorithm for signal processing

RVFL [21] and ELM [22] are similar SLFNs, prevalent for biomedical signal processing due to their compact architecture and fast training and inference. A combination of CNN and RVFL [23] has been employed to classify Alzheimer's disease diagnoses based on MRI and PET images. RVFL has also been utilized as a classifier for Parkinson's disease using transcranial sonography and MRI datasets [24]. ELM and its variants have been applied in the biomedical field for classifying biometric signals, including EEG [25], ECG [26], and PPG [27]. Besides, ELM has also been utilized for parameter reconstruction in single-photon detectors and object classification through heterogeneous fog using a low single-photon avalanche diode (SPAD) array [28]. Also, ELM has been employed for fluorescence lifetime reconstruction in biomarker-functionalized cancer cells, utilizing a photomultiplier and a time-correlated single-photon counting (TCSPC) module [29]. In fluorescence lifetime imaging (FLIM), the current application of ELM [29] demonstrates superior estimation speed while maintaining comparable accuracy to DNNs [30], also exhibits promising fast-training advantages for fluorescence lifetime sensing in flow cytometry [31].

### 2.2. Algorithms for BFI reconstruction

Fitting measured $g_{2}$ to extract BFI and *β* is a challenging and ill-posed regression problem. Various MATLAB NLSF functions, such as *lsqnonlin()* (which employs the interior-reflective Newton method [32]), *fminsearch()* (the Nelder-Mead simplex algorithm [11]), and *optimset()* (the Levenberg-Marquardt method [33]), have been utilized to estimate BFI and evaluate errors arising from uncertainties in optical properties and tissue thickness. The *N^th^*-order Taylor polynomial approximation [34] was introduced to facilitate real-time BFI reconstruction.

Besides fitting, data-driven approaches have gained traction for BFI reconstruction, thanks to precise analytical modeling and MC simulation of $g_{2}$ . A CNN was initially proposed [17] for BFI estimation from simulated $g_{2}$ curves from MCX [35]. A long-short-term memory (LSTM) was firstly employed for real-time continuous BFI monitoring [18]. Recurrent Neural Network (RNN)-types DNNs have been implemented to enhance feature extraction capabilities on 1D data sequences, enabling the inference of absolute BFI [19] and relative BFI (rBFI) [20]. An LSTM-based RNN was proposed [36] to continuously reconstruct rBFI and oxygen saturation for multi-wavelength DCS. Another LSTM [37] was presented to separately estimate BFI values from deep and shallow layers from simulated data and a two-layer phantom. Besides, Wang *et al.* [16] proposed a DNN model that retrieves BFI and β from $g_{2}$ from a three-layer model. Zang *et al.* [14] reported a hardware-friendly DNN for BFI and β reconstruction from a semi-infinite medium, integrating $g_{2}$ generation from intensity data and BFI inference accelerator on FPGA.

In contrast to existing data-driven literature in DCS, the novelty of this work is that we apply RVFL and ELM for BFI estimation. Our approach offers a novel paradigm for real-time training and inference and automated data generation for DCS without using high-end computing resources. This contrasts sharply with previous studies, which necessitate lengthy training times on high-end GPUs and involve complex data generation and collection processes. The compact network topology saves DCS systems’ computational overhead, while the rapid processing enables real-time monitoring in practical applications.

## 3. Problem definition

### 3.1. DCS analytical model

We focus on BFI and the coherence factor β reconstruction from the semi-infinite homogeneous model and the three-layer adult head model for CW-DCS. Our previous study [16] demonstrated that the analytical models generate $g_{2}$ curves consistent with MC simulations. Both the analytical models and the parameters used in the semi-infinite and three-layer media have been elaborated in our previous review [5], and are summarized here in Table 1. *ρ* is the source-detector separation, $\lambda_{DCS}$ is the wavelength, $\mu_{s}^{\prime }$ and $\mu_{a}$ are the reduced scattering and absorption coefficient, *α* is the ratio between dynamic scatters and all scatters, and $z_{0}$ is the distance between the virtual isotropic point source and the tissue surface. $R_{eff}$ is the effective reflection coefficient, which depends on the refractive index of the tissue $n_{0}=n_{tissue}/n_{air}$ . $T_{b}$ is the time interval between two lag times, *m* is the bin index, *t* is the integration time, $n=IT_{b}$ is the average number of photons in a time bin with the photon rate *I*. Γ is the decay rate of $g_{1}$ , obtained by fitting measured or simulated $g_{2}$ according to $g_{2}(\tau )\approx 1+\beta \text{exp}(-2\Gamma \tau )$ . δ(τ) is the noise applied to simulated noiseless $g_{2}(\tau )$ , adopted from [11,38]. $J_{0}$ is the zero*^th^* order Bessel function of the first kind, computed by the MATLAB function *besselj().* *q* is the radial spatial frequency. $L_{l}\;(l=1,\;2,\;3)$ is the depth of Layer 1, 2, and 3. $D_{B(l)}$ is the effective diffusion coefficient in the $l_{th}$ layer.

**Table 1.** Analytical modes of semi-infinite homogenous and three-layer medium.^*a*^^*b*^^*c*^

	Semi-infinite homogenous model	Three-layer human brain model
$G_{1}(\vec{r},\tau )$	$\begin{array}{c} G_{1}(\vec{r},\tau )=\frac{3\mu_{s}^{\prime }}{4\pi }\left(\frac{\exp(-Kr_{1})}{r_{1}}-\frac{\exp(-Kr_{2})}{r_{2}}\right),\\ K=\sqrt{3\mu_{a}\mu_{s}^{\prime }+\alpha \mu_{s}^{\prime 2}k_{0}^{2}\langle \Delta r^{2}(\tau )}\rangle ,\\ k_{0}=2\pi /\lambda_{DCS^{\prime }},\\ r_{1}=\sqrt{\rho^{2}+z_{0}^{2}},\\ r_{2}=\sqrt{\rho^{2}+{\left(z_{0}+2z_{b}\right)}^{2}},\\ z_{0}=1/\mu_{s}^{\prime },\\ z_{b}=\frac{2(1+R_{eff})}{3\mu_{s}^{\prime }(1-R_{eff})},\\ R_{eff}=-1.440n_{0}^{-2}+0.710n_{0}^{-1}\\ +0.668+0.0636n_{0},\\ n_{0}\approx 1.35,\\ \langle \Delta r^{2}(\tau )\rangle =6D_{B}{\tau ,}^{a}\\ \alpha D_{B}=BF{I,}^{b}\\ \alpha \approx 1.^{c} \end{array}$	$\begin{array}{c} Num=3\mu_{s1}^{\prime }z_{0}(\varphi_{1}D_{1}\cosh(\varphi_{1}(L_{1}-z_{s}))\\ \left(\varphi_{2}D_{2}\cosh(\varphi_{2}L_{2})+\varphi_{3}D_{3}\sinh(\varphi_{2}L_{2})\right)\\ +\varphi_{2}D_{2}(\varphi_{3}D_{3}\cosh(\varphi_{2}L_{2})+\varphi_{2}D_{2}\text{sinh}(\varphi_{2}D_{2}))\\ \text{sinh}(\varphi_{1}(L_{1}-z_{s}))),\\ Denom=\varphi_{2}D_{2}\cosh(\varphi_{2}L_{2})(\varphi_{1}(D_{1}\\ +\varphi_{3}D_{3}z_{0})\text{cosh}(\varphi_{1}D_{1})\\ +(\varphi_{3}D_{3}+\varphi_{1}^{2}D_{1}z_{0})\text{sinh}(\varphi_{1}D_{1}))\\ +(\varphi_{1}(\varphi_{3}D_{1}D_{3}+\varphi_{2}^{2}D_{2}^{2}z_{0})\\ \text{cosh}(\varphi_{1}D_{1})+(\varphi_{2}^{2}D_{2}^{2}+\varphi_{1}^{2}\varphi_{3}D_{1}D_{3}z_{0})\\ \text{sinh}(\varphi_{1}D_{1}))\text{sinh}(\varphi_{2}L_{2}),\\ \varphi_{l}=3\mu_{a(l)}\mu_{s(l)}^{\prime }+6k_{0}^{2}\mu_{s(l)}^{\prime 2}D_{B(l)}\tau +q^{2},\;l=1,\;2,\;3,\\ {\hat{G}}_{0}(q,z,\tau )=\frac{Num}{Denom},\\ G_{0}(r,\tau )=\frac{1}{2\pi }\int dq{\hat{G}}_{0}(q,\tau )qJ_{0}(\rho q) \end{array}$
$g_{1}(\rho ,\tau )$	$g_{1}(\rho ,\tau )=\frac{G_{1}(\rho ,\tau )}{G_{1}(\rho ,\tau =0)}$	$g_{1}(\rho ,\tau )=\frac{G_{0}(r,\tau )}{G_{0}(r,\tau =0)}$
$g_{2}(\rho ,\tau )$	$g_{2}(\rho ,\tau )=1+\beta g_{1}{\left(\rho ,\tau \right)}^{2}$	
Noise model	$\delta (\tau )=\sqrt{\frac{T_{b}}{t}}{\left[\beta^{2}\frac{(1+e^{-2\Gamma T_{b}})(1+e^{-2\Gamma \tau })+2m(1-e^{-2\Gamma T_{b}})e^{-2\Gamma \tau }}{1-e^{-2\Gamma T_{b}}}+2{\left\langle n\right\rangle }^{-1}\beta (1+e^{-2\Gamma \tau })+{\left\langle n\right\rangle }^{-2}(1+e^{-2\Gamma \tau })\right]}^{1/2}$	

^*a*^ $\left\langle \Delta r^{2}(\tau )\right\rangle$ can be approximated to $6D_{B}\tau$ according to Brownian motion model [39].

^*b*^ $\alpha D_{B}$ is estimated as a single parameter as BFI for DCS [40,41].

^*c*^ α≈1 for liquid phantom [41].

### 3.2. ELM and RVFL

For ELM, the weights between the input layer (IL) and hidden layer (HL) are randomly assigned, while the weights between the HL and output layer (OL) are trained. In contrast, RVFL differs by directly connecting the input nodes (IN) not only to the hidden nodes (HN) with random weights and biases but also directly to the output nodes. The weights between the hidden and output nodes in RVFL are trained. The topology of RVFL is depicted in Fig. 1(c). Suppose the training targets for both ELM and RVFL are $y_{i}=[y_{i1},\;y_{i2},\ldots ,y_{im}]\in R^{m}$ , where 1≤i≤N , and *N* is the number of pairs of input data and labels. Suppose the input training samples $x_{i}=[x_{i1},\;x_{i2},\ldots ,x_{in}]\in R^{n}$ . *L* is the number of HNs.

The computational properties of ELM and RVFL are summarized in Table 2, where ⟨⋅⟩ indicates the inner product. $\sigma_{k}$ is the bias parameter of *k^th^* HN. ω is the matrix of parameters to be trained, connecting the HL and OL. The activation function θ is usually *sigmoid()*, *sine()*, and *RBF()*. $w_{k}$ is the weight vector connecting INs and i*^th^* HN. *I* is an identity matrix with a dimension of (n+L,n+L) . ξ is the regularization hyper-parameter. $H_{ELM}^{†}$ indicate the Moore-Penrose pseudoinverse of matrix of $H_{ELM}$ , implemented using *pinv()* in MATLAB.

**Table 2.** Summary of computing of ELM and RVFL.

	ELM	RVFL
Training input	$x_{i}=[x_{i1},\;x_{i2},\ldots ,x_{in}]\in R^{n},1\leq i\leq N$	
Training target	$y_{i}=[y_{i1},\;y_{i2},\ldots ,y_{im}]\in R^{m},1\leq i\leq N$	
Weight between IL and HL	$w_{k}={\left[w_{k1,\;}\;w_{k2,\;}\ldots ,\;w_{kn,\;}\right]}^{T},1\leq k\leq L$	
Weight between HL and OL	$\begin{array}{c} \omega ={\left[\omega_{1},\;\omega_{2},\ldots ,\;\omega_{L}\right]}_{L\times m}^{T},\\ \omega_{k}=[\omega_{k1},\;\omega_{k2},\ldots ,\;\omega_{km}] \end{array}$	$\begin{array}{c} \omega ={\left[\omega_{1},\;\omega_{2},\ldots ,\;\omega_{n+L}\right]}_{(n+L)\times m}^{T},\\ \omega_{k}=[\omega_{k1},\;\omega_{k2},\ldots ,\;\omega_{km}] \end{array}$
Forward model Hω=Y	$f(x_{i})=\overset{L}{\underset{k=1}{\sum }}\omega_{k}\theta (w_{k},x_{i}+\sigma_{k})$	$f(x_{i})=\overset{n}{\underset{k=1}{\sum }}\omega_{k}x_{ik}+\overset{L}{\underset{k=n+1}{\sum }}\omega_{k}\theta (w_{k},x_{i}+\sigma_{k})$
HL output matrix	$H_{ELM}={\left[\left(\begin{array}{ccc} \theta (w_{1}x_{1}+\sigma_{1}) & \cdots & \theta (w_{L}x_{1}+\sigma_{L})\\ \vdots & \ddots & \vdots \\ \theta (w_{1}x_{N}+\sigma_{1}) & \cdots & \theta (w_{L}x_{N}+\sigma_{L}) \end{array}\right)\right]}_{N\times L}$	$\begin{array}{c} H_{1}={\left[\begin{array}{ccc} \begin{array}{cc} x_{11} & x_{12} \end{array} & \cdots & x_{1n}\\ \vdots & \ddots & \vdots \\ \begin{array}{cc} x_{N1} & x_{N2} \end{array} & \cdots & x_{Nn} \end{array}\right]}_{N\times n},\\ H_{2}={\left[\left(\begin{array}{ccc} \theta (w_{1}x_{1}+\sigma_{1}) & \cdots & \theta (w_{L}x_{1}+\sigma_{L})\\ \vdots & \ddots & \vdots \\ \theta (w_{1}x_{N}+\sigma_{1}) & \cdots & \theta (w_{L}x_{N}+\sigma_{L}) \end{array}\right)\right]}_{N\times L},\\ H_{RVFL}={\left[H_{1}H_{2}\right]}_{N\times (n+L)} \end{array}$
Training ω	$\omega =H_{ELM}^{†}Y$	$\omega =\left\{ \begin{array}{c} {\left(H_{RVFL}^{T}H_{RVFL}+\lambda I\right)}^{-1}H_{RVFL}^{T}Y,\;\;(n+L)\leq N\\ H_{RVFL}^{T}{\left(H^{T}H_{RVFL}+\lambda I\right)}^{-1}Y,\;\;N<(n+L) \end{array}\right.$

### 3.3. Apply analytical models to training and test dataset generation

We generated $g_{2}$ samples to train the RVFL and ELM models using analytical models of semi-infinite and three-layer tissue as described in Table 1. Each sample was calculated using 128 logarithmically spaced lag times, ranging from 10^−7^ to 10^−1^ seconds. Corresponding labels of each $g_{2}$ curve are BFI and *β*. The evaluation baseline was set at *L* = 500, with *N* = 5,000, *n* = 128, *m* = 2, referring to the variables in Table 2. Additionally, 500 independent $g_{2}$ curves were generated for testing. For the semi-infinite model dataset generation, $\mu_{s}^{\prime }=1.6\;{\text{mm}}^{-1}$ and *μ_a_* = 0.0027 mm^−1^ were adopted from [13] to mimic blood flow in living tissue. For the three-layer model, $\mu_{s1}^{\prime }=0.635\;{\text{mm}}^{-1}$ , $\mu_{s2}^{\prime }=0.851\;{\text{mm}}^{-1}$ , $\mu_{s3}^{\prime }=1.099\;{\text{mm}}^{-1}$ and $\mu_{a1}=0.019\;{\text{mm}}^{-1}$ , $\mu_{a2}=0.014\;{\text{mm}}^{-1}$ , $\mu_{a3}=0.019\;{\text{mm}}^{-1}$ , along with thickness of 5 mm, 7 mm, ∞ , for the scalp, skull, and brain layers, respectively, adopted from [16,42]. Notably, although previous literature [16,17] used semi-infinite datasets to train neural networks, the accuracy of cerebral blood flow index (CBFI) and relative CBFI (rCBFI) reconstructions were confounded by extracerebral layers. In this work, we investigate the error by applying semi-infinite-based neural networks (RVFL, ELM, and CNN) and fitting algorithms (*fminsearch()*, *lsqnonlin()*, and *lsqcurvefit()*, implemented in MATLAB) to analyze three-layer datasets. For the two types of datasets with common experimental settings, ρ=25mm , *λ_DCS_* = 785 nm, *n*_0_ = 1.35. Noise parameters, *I* and *T* shown in Table 1, were randomly sampled between [1.5 × 10^4^, 2.5 × 10^4^] counts per second (cps) and between [5, 25] seconds, respectively. Figure 1(a) and 1(b) provide an overview of the optical properties and layer thicknesses of the semi-infinite homogenous and three-layer brain models used in both MC and analytical simulations. Figure 1(c) illustrates the data flow and RVFL topology.

## 4. Performance evaluation

### 4.1. Evaluation on analytical datasets on semi-infinite and three-layer models

This section evaluates the accuracy and precision of RVFL using datasets generated from the analytical semi-infinite and three-layer models. For a fair comparison with standard algorithms, we also implemented three optimization-based fitting methods and a CNN.

**Fig. 2.**
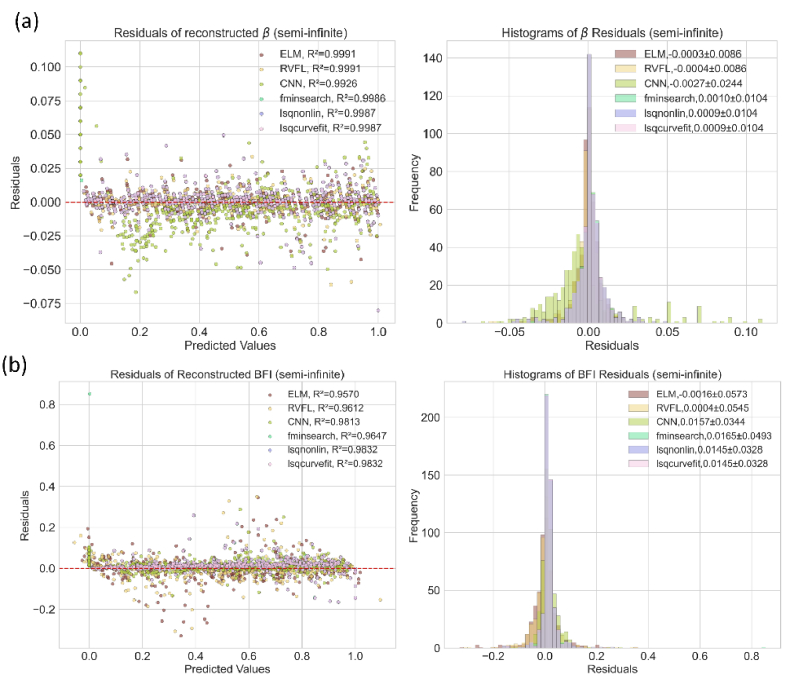
Reconstructed distributions of (a) BFI and (b) β from independent semi-infinite test datasets using algorithms implemented (or trained) with the semi-infinite model.

**Fig. 3.**
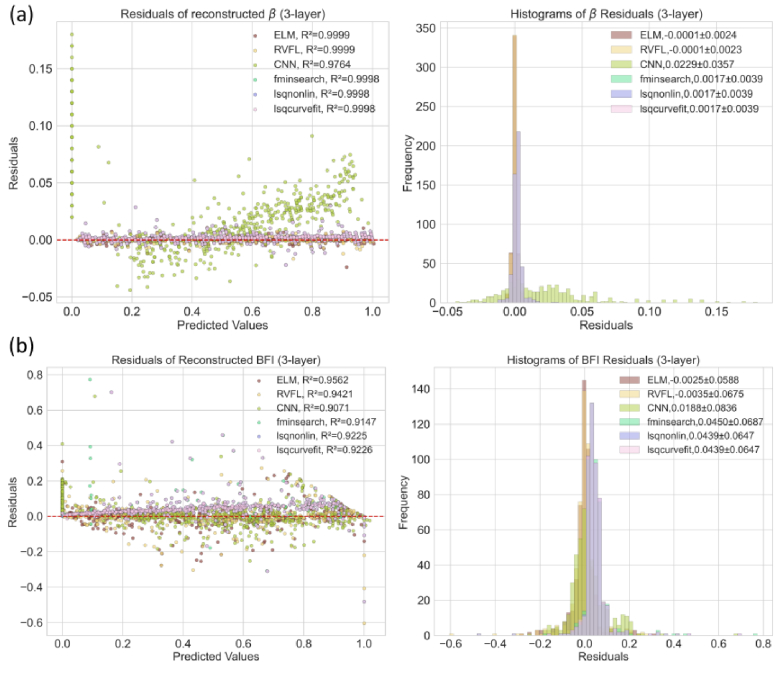
Reconstructed distributions of (a) BFI (CBFI for Layer-3) and (b) β from independent three-layer test datasets using algorithms implemented (or trained) with the three-layer model.

In Fig. 2 and Fig. 3, we visualize the reconstructed BFI and *β* from independent test datasets generated by the semi-infinite and three-layer models. For the residuals of *β* reconstruction in Fig. 2(a), both ELM and RVFL show higher accuracy than CNN and fitting methods, as reflected by *R^2^*. Although the CNN was trained on the same datasets as ELM and RVFL, its *R^2^* is lower, with a few noticeable outliers. This is because the hyperparameters of the CNN model, such as learning rate, loss function, and topology, were not fully optimized for fair comparisons. The performance of CNN can be enhanced by fine-tuning the parameters. The three fitting methods exhibit similar *R^2^* values. Additionally, we plot the residual distributions for all algorithms. ELM and RVFL demonstrate smaller absolute means $\left(\mu_{abs}\right)$ and lower standard deviations $\left(\sigma_{std}\right)$ than CNN and the fitting methods. In terms of BFI reconstruction, shown in Fig. 2(b), *lsqcurvefit()* and *lsqnonlin()* present higher *R^2^* than *fminsearch()*, among the three fitting methods. The two fitting methods also slightly outperform ELM and RVFL regarding *R^2^*. CNN achieves the highest *R^2^* among the three data-driven methods. Regarding the histogram of residuals in Fig. 2(b), RVFL achieves the highest accuracy (smallest $\mu_{abs}$ ) but slightly lower precision (larger $\sigma_{std}$ ) than CNN and the fitting methods.

Unlike our previous study [16], where a DNN model was trained using a semi-infinite-based dataset to infer BFI for a three-layer $g_{2}$ , we trained ELM, RVFL, and CNN models separately using both semi-infinite and three-layer datasets. This approach aims to enhance BFI estimation in deep tissue covered by superficial layers. Figure 3 depicts the performance on three-layer datasets. For *β* reconstruction, the fitting algorithms, ELM, and RVFL achieve higher accuracy than CNN. Similarly, ELM and RVFL present the highest accuracy $\left(\mu_{abs}\right)$ and precision $(\sigma_{std})$ compared to the others. For BFI (Layer-3) reconstruction, ELM and RVFL also outperform the other models with higher *R^2^* and smaller $\mu_{abs}$ . The *lsqnonlin()* and *lsqcurvefit()* algorithms achieve nearly the same *R^2^*, which is higher than that of *fminsearch()*. Like the evaluation on semi-infinite datasets, CNN is not fully optimized for a fair comparison and still holds potential for performance enhancement. For both *β* and BFI reconstruction, ELM and RVFL produce overall slightly smaller predicted values, as indicated by negative $\mu_{abs}$ values. In contrast, CNN and the fitting algorithms produce slightly larger predicted values, as indicated by positive $\mu_{abs}$ values. This phenomenon also occurs in the evaluation on semi-infinite datasets, as shown in Fig. 2.

For clearer visualization of the regression performance of each algorithm on semi-infinite and three-layer test datasets, quantity-versus-quantity (Q-Q) plots are presented in Fig. S1 and Fig. S2 in the Supplement 1.

### 4.2. Evaluation of in-silico datasets on semi-infinite and three-layer models

This section investigates the performance differences between two RVFL models—semi-RVFL and tl-RVFL—trained using semi-infinite and three-layer datasets, respectively, for processing MC-three-layer datasets. A previous study [15] explored the accuracy of a fitting method based on the three-layer analytical model, which tested clear MC-simulated $g_{2}$ curves. However, real $g_{2}$ data is always subject to noise. Therefore, in this work, noise was added to the MC-generated $g_{2}$ according to the noise model described in Table 1. Optical parameters mentioned in Section 3.3 were implemented in a MATLAB script. The generation process for $500\;g_{2}$ curves takes approximately 30 minutes with GPU acceleration on an NVIDIA RTX 2000 Ada, with an initialized photon count of 10^7^.

**Fig. 4.**
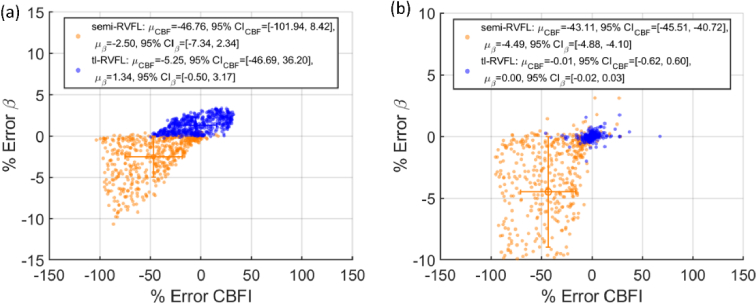
Evaluation of semi-RVFL and tl-RVFL, tested with (a) three-layer MC datasets and (b) three-layer analytical simulation datasets.

As existing literature [6,17,43] assumes the tissue under test is a one-layer semi-infinite homogeneous model for simplicity, estimation errors are induced when testing tissues with complex geometries, such as the brain or arm. In this study, we introduce fast dataset generation and neural network training pipelines that enhance accuracy while preserving simplicity. Therefore, we also compared the performance of the semi-RVFL and tl-RVFL models, trained with analytical semi-infinite and three-layer datasets, respectively, to estimate outcomes on independent MC-generated three-layer datasets. The values of *β* and CBFI for both models in the test datasets were randomly assigned from the ranges [0.01, 1], and [10^−8^, 10^−5^] mm^2^/s, respectively. Figure 4 depicts the scatter plots of Error%, the mean of Error% (*μ*), and the 95% confidence interval (CI) for both. For MC-simulation in Fig. 4(a), tl-RVFL yields higher accuracy for CBFI and *β* reconstruction than semi-RVFL, as indicated by smaller absolute *μ* and CI. Similarly in the analytical simulation shown in Fig. 4(b), the accuracy of *β* and CBFI reconstruction from tl-RVFL significantly surpasses that of semi-RVFL. These results indicate that using semi-RVFL leads to substantial errors in three-layer media, whereas applying the three-layer analytical model enhances reconstruction accuracy.

**Fig. 5.**
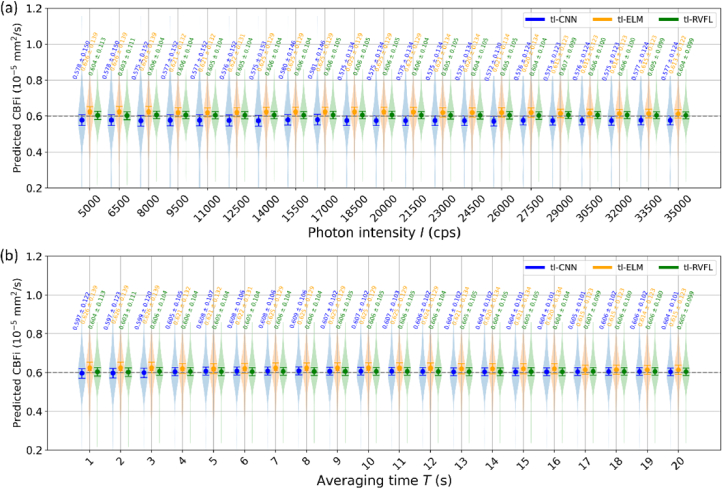
CBFI reconstruction from three data-driven algorithms trained by three-layer MC simulated data, evaluated by (a) *I*- and (b) *T*-controlled noise added to $g_{2}$ . Solid dots and bars in each violin are means and 95% CI.

**Fig. 6.**
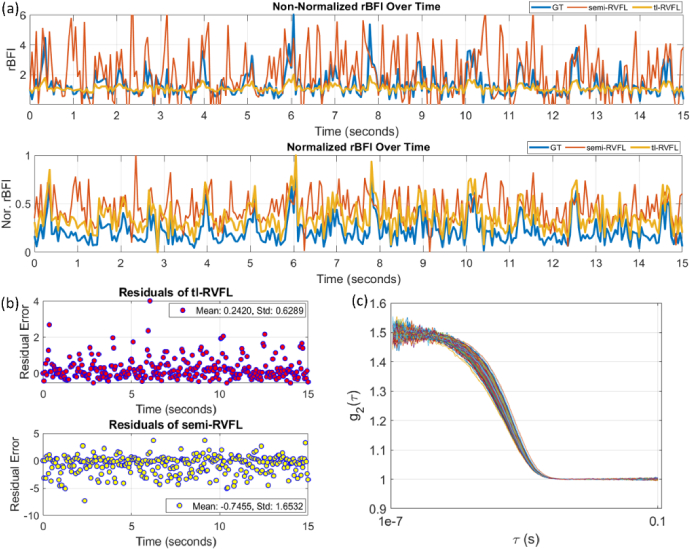
rBFI evaluation. (a) 301 rBFI points over 15 seconds from the public datasets, including GT rBFI (blue line), estimated rBFI from semi-RVFL (red line), and tl-RVFL (yellow line). (b). Residual errors of reconstructed rBFI values from semi-RVFL and th-RVFL, annotated with mean and std. (c). Generated noisy $g_{2}$ curves using MCX from the GT BFI values in the datasets.

Since tl-RVFL demonstrates superior BFI reconstruction than semi-RVFL, we further evaluated the three-layer model, under various noise levels. For this evaluation, we included tl-RVFL, tl-ELM, and tl-CNN. The CNN architecture was adopted from a previous study [14], utilizing the same hyperparameters and topology. Identical to the previous study [14], an early-stop function with 35 patient epochs was integrated during training. This saved the pre-trained model with the smallest validation error to prevent overfitting. According to the noise model in Table 1, the noise’s magnitude can be controlled by photon intensity *I* and total averaging time *T*. We set *I* and *T* with a range of [5,000, 35,000] with 20 intervals ( $100\;g_{2}$ curves per interval), and *T* within [1, 20] seconds with 20 intervals (100 curves per interval), respectively. *T* and *I* were fixed to 20 s and 25,000 cps for evaluation of *I* and *T*, respectively. BFIs and *β* for three layers were fixed at 10^−6^, 0, 6 × 10^−6^ mm^2^/s, and 0.5 for *T*- and *I*-oriented noise evaluation. As shown in Fig. 5(a), tl-CNN and tl-ELM exhibit lower and higher means of reconstructed CBFI across all *I* values, respectively. Additionally, tl-CNN demonstrates higher standard deviations (*std*) than tl-ELM and tl-RVFL. The *stds* converge as *I* increases. Interestingly, the means of tl-RVFL remain stable across all *I* values, whereas the means of tl-ELM and tl-CNN show a converging trend toward the ground truth (GT) value as *I* increases. Among the three models, tl-RVFL achieves the highest mean accuracy and the smallest *stds* across all *I* values. In the evaluation of *T*-controlled noise, shown in Fig. 5(b), tl-CNN and tl-RVFL display similarly accurate CBFI reconstruction, while tl-ELM exhibits a higher mean than both. Unlike Fig. 5(a), tl-ELM shows the highest *stds* across different *I* values.

### 4.3. Relative BFI evaluation

We adopted an open-source BFI waveform [44] from an adult for forehead sensing to evaluate the applicability of our model in realistic rBFI reconstruction. Using the BFI values from the waveform as a reference, we generated $g_{2}$ curves using MCX [35] and the noise model in Table 1. The dataset from the literature [44] consists of 44 windows of trials, each containing 301 BFI data points. For testing, we selected one of these windows. The rBFI equation is given by $rBFI=\frac{BFI}{BFI_{0}}$ , where $BFI_{0}$ is the initial BFI (7.75 × 10^−6^ mm^2^/s) in the window. The 301 BFIs were used to generate $g_{2}$ curves with fixed β=0.5 . The reference rBFI (blue lines) are depicted in Fig. 6. In Fig. 6(a), the estimated rBFI waveform from the tl-RVFL (shown in yellow) exhibits a similar fluctuation pattern to the GT. However, there is a notable difference in magnitude between the two waveforms. The GT BFIs were retrieved using a semi-infinite homogeneous fitting model from the literature [44], which did not account for the interference of superficial layers. In contrast, tl-RVFL was trained with a three-layer model that includes the effects of the scalp and skull layers, resulting in subtle changes in the rBFI. Despite these differences, the rBFI waveform retrieved from tl-RVFL closely mirrors the changes observed in the GT. For better visualization, we have shown the normalized rBFI waveforms accordingly in Fig. 6(a). Conversely, the semi-RVFL model demonstrates inaccurate waveforms, with lower accuracy and precision, as illustrated in Fig. 6(a) and (b), respectively. The MC-simulated $301\;g_{2}$ curves derived from the BFI points in the public datasets are depicted in Fig. 6(c).

**Fig. 7.**
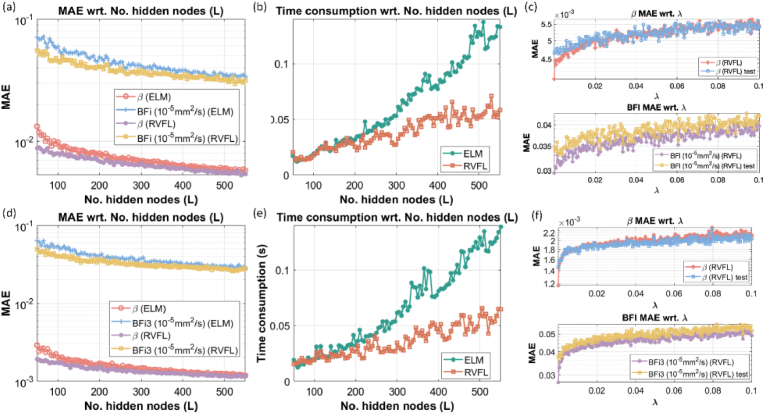
Performance evaluation of ELM and RVFL for (a-b) semi-infinite and (d-e) three-layer model wrt. *L*, in terms of MAE and training time consumption. (c) and (f) show the relationship between accuracy and λ for training and test datasets.

**Fig. 8.**
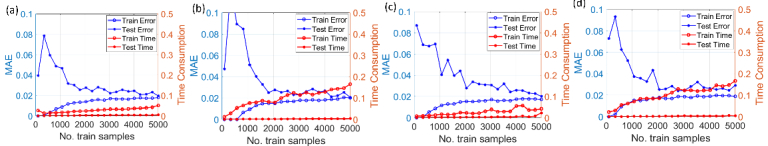
Performance evaluation of accuracy versus the number of training samples was conducted on semi-infinite test datasets for (a) RVFL and (b) ELM, respectively. Similarly, the evaluation was performed on three-layer test datasets for (c) RVFL and (d) ELM, respectively.

### 4.4. Computational evaluations

We explored the influence of tuning the hyper-parameters of RVFL for accuracy and computing speed, providing the optimal combination. Firstly, we evaluated the computational performance and training accuracy of BFI and *β*. Also, we assessed the effect of the hyperparameter inference. As shown in Fig. 7(a), we investigated how *L* affects the three-layer model’s training accuracy for BFI and *β*. For a small *L*, RVFL presents higher accuracy. As *L* increases, ELM and RVFL converge to a similar MAE, although RVFL still exhibits slightly higher accuracy than ELM. We further explored the relationship between *L* and computing time, shown in Fig. 7(b). Figure 7(a) and (d) also reflect that there is no overfitting issue in our cases. Because ELM and RVFL training use matrix inversion, overfitting can occur when *L* is unnecessarily large [45]. We used *L* = 500 for all test cases, which is smaller than the maximum *L* in Fig. 7(a) and (b), where no accuracy degradation occurs until *L* = 550. Therefore, overfitting is avoided. The time consumption of ELM grows faster than RVFL when *L* increases. This is primarily because RVFL involves only matrix multiplications and division during training and inference. In contrast, ELM requires the inversion of a large pseudo-matrix, which typically involves QR or singular value decomposition. The accuracy and time consumption for the three-layer test datasets, as affected by *L*, shown in Fig. 7(d) and (e), exhibit a similar trend and comparison to the semi-infinite test datasets. In RVFL, the hyper-parameter *λ* prevents overfitting yet affects RVFL's accuracy. We investigated the effect of varying *λ* on accuracy for training and test datasets. As shown in Fig. 7(c), the range of *λ* in our test is [1 × 10^−6^, 0.1]. The MAE for both training and testing for BFI (Layer-3) and *β* increases as *λ* increases. The MAE is the smallest when $\lambda =10^{-6}$ . In Fig. 7(c) and (f), although, the MAEs of the reconstructed *β* from three-layer datasets are overall smaller than the semi-infinite case, the MAEs of the reconstructed BFI for three-layer datasets are around 3-fold higher than the semi-infinite case.

We also investigated how the size of training datasets affects training and test accuracy, as well as time consumption. The range of the number of training datasets (#Tr) is [100, 5000], divided into 20 intervals. Since there is no validation process during the training of RVFL and ELM, the number of test datasets (#Te) for each interval is set to 10% of the corresponding training datasets. Starting with the evaluation of semi-infinite datasets, as shown in Fig. 8(a), there is significant underfitting during the first eight intervals, where the training MAE is very small while the test MAE is large and does not converge until the 9^th^ interval. The MAE is calculated as the sum of the predicted BFI and *β*. Compared to RVFL, ELM exhibits more pronounced underfitting, as indicated by a higher MAE before the 8^th^ interval, and ELM’s time consumption increases more rapidly as #Tr increases. For the three-layer test datasets, as shown in Fig. 8(b), a similar underfitting issue is observed during the first eight intervals. Therefore, selecting #Tr = 5,000 is appropriate for our training cases, as it demonstrates convergence between the training and test MAE.

Table 3 compares the existing DNN-based BFI reconstruction methods in terms of FLOPs, number of parameters (# Param.), number of layers (# Layers), training time with the number of samples used in the literature, inference time per sample, and $\rho^{\prime }\text{s}$ reconfigurability. Although Nakabayashi *et al.*’s model [37] used the fewest samples, the length of each $g_{2}$ curve is 501, longer than 128 in our $g_{2}$ curves. Therefore, RVFL and ELM equivalently require the smallest volume of training samples, potentially reducing the time required for data generation and collection. RVFL achieves faster training times than other methods, even with less powerful computing hardware. Apart from [16] and [37], the *ρ* values in the other studies are fixed during inference and require re-training if the environment setup changes. Thanks to its fast training, RVFL can swiftly adjust to new setups. Although LSTM [18] has the fewest #FLOPs and #Param., its inference time is slower than our methods. Additionally, unlike regularized matrix multiplication and additions, LSTM blocks involve several skip connections, and the data dependency hinders the parallelization on embedded hardware. Between RVFL and ELM, RVFL is faster than ELM in training yet slower than ELM in inference. RVFL shows smaller MAEs than ELM in training and inference.

**Table 3.** Comparisons of existing DNN models for BFI estimation.

DNN model	FLOPs	#Param.	#Layers	Training performance	Inference performance^*b*^	ρ (mm)
RNN [19], 2019	∼1,050,624,000	174,080	20	N/A	N/A	25
CNN [17], 2020	589,174,520	2,200,320	161	∼30.5 h, $72,000\;g_{2}$ (NVIDIA GeForce GTX 1080 TI)	0.46 ms	27.5
LSTM [18], 2021	5,120	1,161	2	N/A, $6,000\;g_{2}$ Intel Core i7-4700MQ CPU	19.95 ms	15
ConvGRU^*a*^ [20], 2023	∼165,888	∼ 2,688	10	N/A (Intel Core i7-6700MQ CPU)	1.31 ms	20
DCS-NET [16], 2023	4,911,904	25,506	18	∼13 min, $200,000\;g_{2}$ (NVIDIA Quadro RTX 5000)	1.03 ms	Reconfigurable
LSTM [37], 2023	∼3,488,768	∼3,492,866	2	N/A, Nvidia K80, $3,618\;g_{2}$	N/A	Reconfigurable
LSTM-RNN [36], 2024	43,040	16,912	3	N/A	0.056 ms	15
**This work, ELM**	129,000	65,500	**1**	**0.488 s (Intel-i5 3.1 G Hz),** **5,000** $g_{2}$ , **MAE** ^*c*^ **: 0.0153**	**0.026 ms,** **MAE** ^*c*^ **: 0.0361**	Reconfigurable
**This work, RVFL**	129,521	65,756	**1**	**0.191 s (Intel-i5 3.1 G Hz),** **5,000** $g_{2}$ , **MAE** ^*c*^ **: 0.0144**	**0.052 ms,** **MAE** ^*c*^ **: 0.0357**	Reconfigurable

^*a*^ The number of output channels of three convolutional layers is not available in the paper, we assume 16, 32, and 64 are common settings.

^*b*^ Inference time per $g_{2}$

^*c*^ Mean Absolute Error (MAE), summed by MAE of BFI and β .

## 5. Discussion

**Fig. 9.**
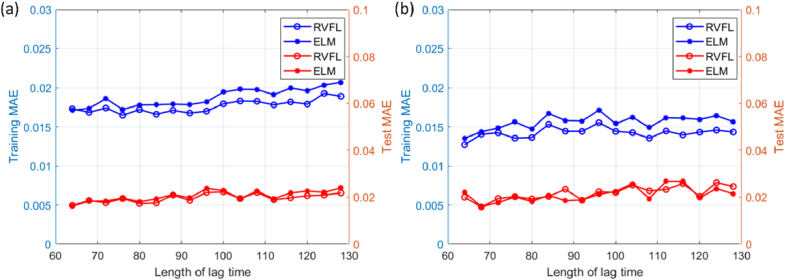
The relationship between accuracy and lag time length was evaluated on RVFL and ELM, tested using (a) a semi-infinite dataset and (b) a three-layer dataset.

This work uses consistent $\mu_{s}^{\prime },\mu_{a}$ , and ρ for training networks rather than using parameter ranges, as we found that training accuracy is significantly affected when parameters vary, apart from BFI and β . The generalization of RVFL is impacted by these consistent parameters ( $\mu_{s}^{\prime },\mu_{a}$ , and ρ ) if the experiment setup alters. To enhance generalization, we linearly assigned 50 values for $\mu_{a}$ and 50 values for $\mu_{s}^{\prime }$ within the ranges of [0.5, 1.6] mm^−1^ and [0.001, 1] mm^−1^, respectively. Additionally, we linearly assigned 10 *ρ* values in the range of [10, 35] mm. For each combination of $\mu_{s}^{\prime }$ and $\mu_{a}$ we assigned 5,000 samples. Therefore, for each ρ , we generated 50 × 50 × 5,000 samples to cover the most common settings. With these standby datasets, we can swiftly re-train the model with the appropriate parameter combination if the experimental setup changes. To evaluate the robustness of RVFL and ELM models to varying lag time lengths—arising from hardware correlators producing different lag time durations—we maintained a constant total lag time range during the analysis, from 10^−7^ to 10^−1^ seconds, while varying the length from 64 to 128 in 20 intervals. As shown in Fig. 9, the training and test accuracy of RVFL and ELM remain unaffected by the lag time length, showing the practical applicability.

Here, we outline three critical areas for future work:

1. Although RVFL running on a CPU already achieves real-time performance, $g_{2}$ curves generated from the hardware autocorrelator still need to be transferred to a PC for post-processing. Almeida *et al.* proposed a parallelized OS-RVFL [46], demonstrating that the training phase can be parallelized into sequential learning for each sample, instead of relying on the pseudo-inversion of a large matrix $H_{RVFL}$ as shown in Table 1. This makes it feasible to implement OS-RVFL directly on hardware and integrate it with the hardware correlator, forming an encapsulated module and reducing the post-processing overhead.
2. Although this work focuses on the semi-infinite and three-layer models for CW-DCS, it would be worthwhile to implement analytical models with different geometries, such as two-layer models, and evaluate RVFL’s accuracy and consistency between analytical results and MC simulations. Furthermore, beyond CW-DCS, applying RVFL to analyze and evaluate time-domain [40] and frequency-domain [47,48] DCS numerically also warrants investigation.
3. We plan to apply data generation and model training pipelines to real phantoms or tissues with characterized optical parameters to evaluate our algorithm in a practical setting.

## 6. Conclusion

This work presents an online training and inference RVFL for BFI and β reconstruction in CW-DCS, addressing the challenges of long training times in DNNs and the low accuracy of NLSF methods. This study highlights the potential of RVFL-based models in clinical DCS systems. Testing semi-RVFL with three-layer data reveals that semi-RVFL is less suitable than tl-RVFL due to lower accuracy and precision. Additionally, we discussed the potential of hardware-embedded RVFL and the integration with hardware correlators, thanks to RVFL’s compact architecture and regularized computations. This hardware integration can further enhance the portability of DCS systems by eliminating the need for post-processing.

## Supplemental information

Supplement 1 Figures S1 and S2 https://doi.org/10.6084/m9.figshare.28406882

## Data Availability

Code and data in this paper can be obtained from the authors upon reasonable request.

## Supplemental document

See Supplement 1 for supporting content.
