# Supplementary material for: Fast blood flow index reconstruction of diffuse correlation spectroscopy using a back-propagation-free data-driven algorithm
Source: Biomed Opt Express. 2025 Feb 26;16(3):1254–69. doi: 10.1364/BOE.549363 (PMC11919341; doi:10.1364/BOE.549363)
Supplement: Supplementary file 1 [file boe-16-3-1254-s001.pdf]

Supplemental Document

Biomedical Optics EXPRESS

Fast blood flow index reconstruction of diffuse correlation spectroscopy using a back-propagation-free data-driven algorithm: supplement

ZHENYA ZANG, MINGLIANG PAN, YUANZHE ZHANG, AND DAVID DAY UEI LI*

Department of Biomedical Engineering, University of Strathclyde, 16 Richmond Street, Glasgow, G1 1XQ, United Kingdom

*David.Li@strath.ac.uk

This supplement published with Optica Publishing Group on 26 February 2025 by The Authors under the terms of the Creative Commons Attribution 4.0 License in the format provided by the authors and unedited. Further distribution of this work must maintain attribution to the author(s) and the published article's title, journal citation, and DOI.

Supplement DOI: https://doi.org/10.6084/m9.figshare.28406882

Parent Article DOI: https://doi.org/10.1364/BOE.549363

FAST BLOOD FLOW INDEX RECONSTRUCTION OF DIFFUSE CORRELATION SPECTROSCOPY USING A BACK-PROPAGATION-FREE DATA-DRIVEN ALGORITHM: SUPPLEMENTAL DOCUMENT

The figures below show individual quantity-versus-quantity (Q-Q) plots for each algorithm, evaluated using the semi-infinite and three-layer test datasets.

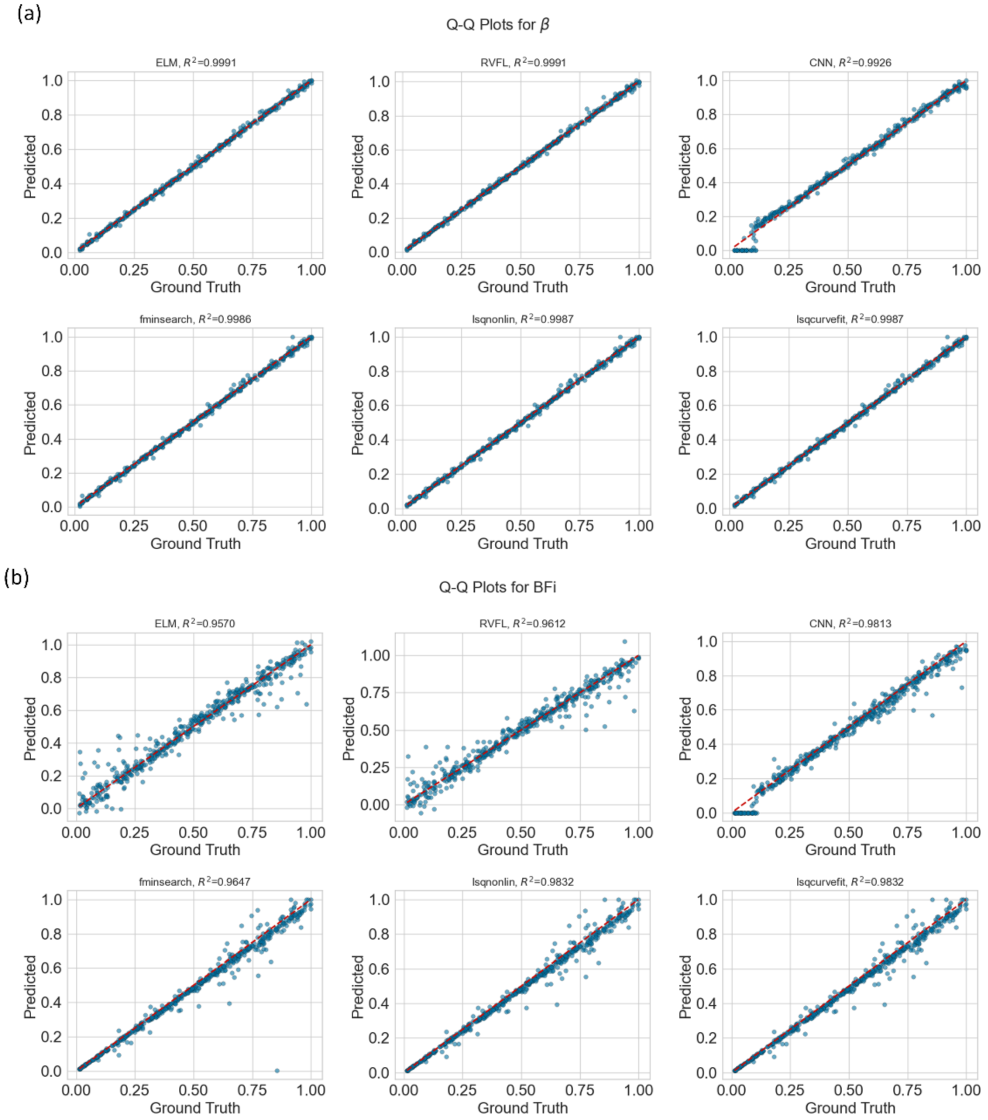

Fig. S1. Individual Q-Q plots of BFI and β from each algorithm were evaluated using semi-infinite test datasets.

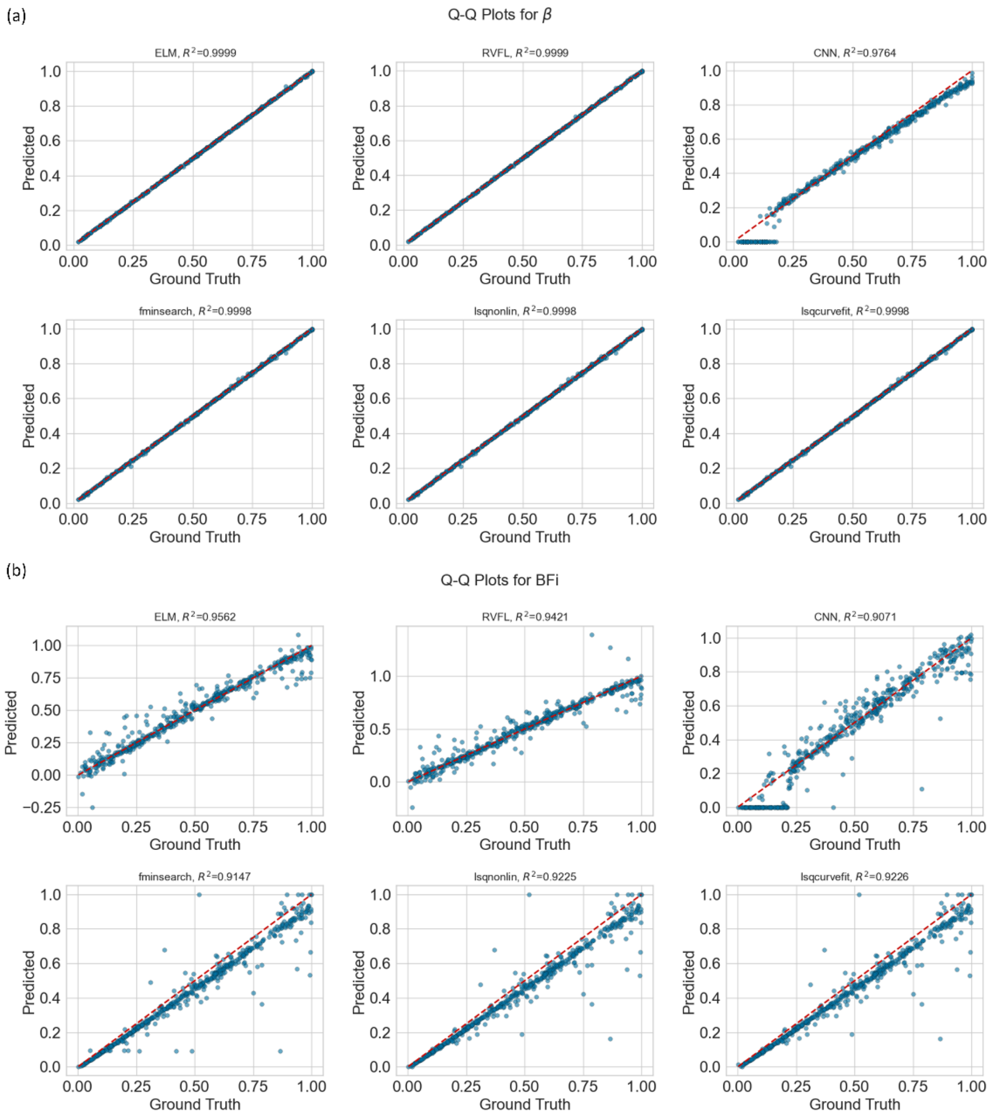

Fig. S2. Individual Q-Q plots of BFI and β from each algorithm were evaluated using three-layer test datasets.
